# Material-induced chondrogenic differentiation of mesenchymal stem cells is material-dependent

**DOI:** 10.3892/etm.2014.1583

**Published:** 2014-02-25

**Authors:** LI ZHENG, JINSONG YANG, HONGSONG FAN, XINGDONG ZHANG

**Affiliations:** 1 Medical and Scientific Research Center, Guangxi Medical University, Nanning, Guangxi 530021, P.R. China; 2 Osteopathy Ward of The First Affiliated Hospital, Guangxi Medical University, Nanning, Guangxi 530021, P.R. China; 3 National Engineering Research Center for Biomaterials, Sichuan University, Chengdu, Sichuan 610064, P.R. China

**Keywords:** chondrogenic differentiation, mesenchymal stem cells, porous materials, biphasic calcium phosphate ceramic, silk fibroin protein matrix, collagen sponge

## Abstract

Certain materials may mimic natural cartilage to provide an amenable cellular microenvironment for the chondrogenic differentiation of mesenchymal stem cells. The chondrogenic differentiation of bone marrow mesenchymal stem cells (BMSCs) has been demonstrated to be induced by collagen-based hydrogels *in vivo*, but whether the induction is material-driven or self-differentiation has not been elucidated. In the present study, BMSCs were encapsulated in porous materials, namely, a biphasic calcium phosphate ceramic (BCP), silk fibroin protein matrix (SFP) and collagen sponge (CS), to further study the chondrogenic effects of various materials. Diffusion chambers that allow the body fluid to permeate and deter the host cells from invasion were also loaded with the cell-scaffold constructs. Chambers containing the scaffold-BMSC composites were implanted subcutaneously in the dorsa of rabbits. The specimens in the chamber were harvested for histological and immunohistochemical analyses eight weeks after implantation. The results showed that no chondrogenic differentiation of the BMSCs occurred when the BMSCs were encapsulated in BCP, SFP and CS, indicating that chondrogenesis induced by materials is material-dependent and that these particular porous materials are not suitable for inducing chondrogenesis. However, the diffusion chamber was effective in preventing host immune rejection, host cell invasion and vascular invasion. The results are likely to serve as a valuable clinical reference when selecting an appropriate scaffold for cartilage repair.

## Introduction

Damaged articular cartilage has a poor capability for self-repair due to the low cell density (1–3). Traditional surgical treatments for cartilage defects are unsatisfactory, and are predominantly hampered by limited resources (2,4). Showing significant promise as viable options, tissue-engineering strategies have been widely studied for cartilage restoration (5). Scaffolds play a crucial role in cartilage tissue engineering. It has been reported that scaffolds that have the ability to induce chondrogenesis are preferably for use in the repair of cartilage defects (6). In a previous study, it was shown that the chondrogenic differentiation of bone marrow mesenchymal stem cells (BMSCs) may be induced by collagen-based hydrogels *in vivo* (7).

However, due to limitations in the variety of material types that have been investigated, the induction of chondrogenesis by a material alone remains disputable. Whether the induction is material-driven or self-differentiation requires full investigation. Furthermore, if the induction is triggered by a material, the question of whether it is material-dependent must also be considered. In the present study, certain types of material that are different from the previously tested hydrogels, namely, a biphasic calcium phosphate ceramic (BCP), silk fibroin protein matrix (SFP) and collagen sponge (CS), were applied to further investigate chondrogenic induction *in vivo* in order to evaluate the significance of the material in chondrogenesis.

## Materials and methods

### Preparation of materials and diffusion chamber

The *in vivo* model used to study the chondrogenic effects of the porous materials was as described previously (2). BCP, SFP and CS were generous gifts from Dr B. Li and from Suzhou University (Suzhou, China), respectively.

The diffusion chamber was made of ultra-high molecular weight polyethylene, with an outer diameter of 10 mm, inner diameter of 6 mm and a height of 5 mm. Each chamber was sealed with a membrane filter (pore size, 0.22 μm) using an adhesive sealant. Prior to the experiment, only one side of each chamber was closed. The chambers were sterilized with γ-irradiation.

### BMSC isolation and culture

Under sterile conditions, newborn rabbits were sacrificed by an overdose of pentobarbital. The bilateral femurs were dissected with the proximal and distal ends snipped off. The bone marrow tissue was flushed out using a 1-ml sterile syringe with minimum essential medium Eagle α modification (α-MEM; Gibco-BRL, Gaithersburg, MD, USA) containing 10% fetal calf serum and antibiotics (100 U/ml penicillin and 100 U/ml streptomycin). Following centrifugation at 800 rpm for 5 min, the cell pellets were collected and resuspended in fresh culture medium. Cells (1×10^5^) were cultured in a 25-cm^2^ culture flask at 37°C and 5% CO_2_. The culture medium was changed every two or three days. The cells of the third passages were harvested for seeding in the scaffold.

### Cell seeding experiment

**Figure 1:**
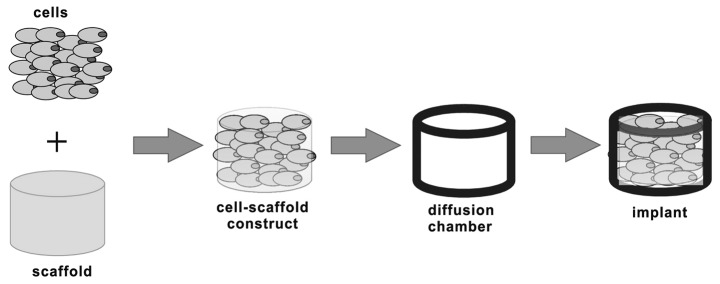
Schematic diagram depicting the preparation of the samples.

The samples for implantation were prepared as shown in Fig. 1. Briefly, when the cells of the third passage reached 80–90% confluency, they were trypsinized using 0.25% trypsin and counted using a hemocytometer. Cell viability was confirmed to be >95% prior to encapsulation. Following centrifugation, the cell pellets were resuspended and seeded in BCP, SFP and CS with a cell density of 2×10^7^ cells/cm^3^. Subsequently, the cell-scaffold composites were enclosed in the diffusion chambers with the previously open side sealed by a membrane filter. The filters on the two sides allow body fluid to pass through while preventing host invasion.

### In vivo experiment

The diffusion chambers carrying the cell-scaffold composites were surgically inserted subcutaneously into a pocket in the dorsa of six-month-old rabbits under sodium barbital anesthesia. The study was conducted in accordance with the US guidelines for laboratory animal use and care (National Institutes of Health publication no. 85-23, revised in 1985). Prior to implantation, leakage of the chambers was carefully checked for and the chambers that failed the check were discarded. Following surgery, the wounds were carefully rinsed with 0.9% saline solution and closed with sutures. All rabbits received ampicillin for two consecutive postoperative days.

### Histological and immunohistochemical staining

Eight weeks after implantation, the specimens in the chamber were harvested for histological and immunohistochemical analyses. The rabbits were sacrificed by an intravenous injection of euthanasia solution, and the scaffold-cell constructs inside the chambers were removed for analysis. Breakage of the chamber was checked for once more. One section of the harvested tissue from the chamber was fixed immediately in 10% aqueous formalin solution overnight, and then dehydrated in a gradient ethanol series, embedded in paraffin and sectioned (5-μm thick). The cross-sections were stained with hematoxylin and eosin (H&E), safranine-O and toluidine blue. The type II collagen was immunohistochemically stained using a monoclonal antibody to type II collagen (no. AF5710; Acris Antibodies GmbH, Hereford, Germany) according to the manufacturer’s instructions.

## Results

### Macroscopic observations

**Figure 2:**
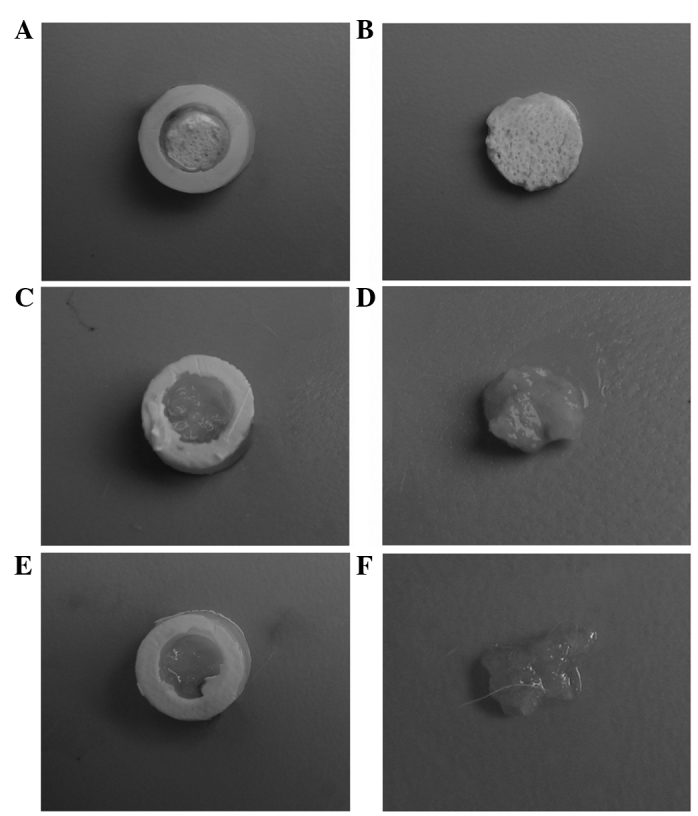
Macroscopic appearance of the specimens (A, C and E) inside the chamber and (B,D and F) out of the chamber following implantation for eight weeks. Fibrous tissue was observed over the (A and B) BCP and (C and D) SFP scaffolds. (E and F) No tissue was observed in the CS scaffold. BCP, biphasic calcium phosphate ceramic; SFP, silk fibroin protein matrix; CS, collagen sponge.

Eight weeks after implantation, the scaffold/cell constructs inside the chambers were harvested. No leakage of the chamber and no vascular invasion were observed for any of the harvested samples, indicating the effectiveness of the diffusion chamber in avoiding a host reaction. An image of a representative sample from each group is shown in Fig. 2. In the BCP and SFP groups, a thin, fibrous tissue, rather than cartilage-like tissue, was formed at the surface of the scaffold. In the CS group, the constructs had almost collapsed and no tissue was observed.

### Histological and immunohistochemical staining

**Figure 3:**
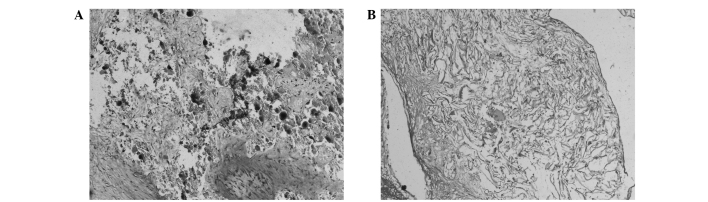
H&E staining of sectioned tissue inside the chamber. Spindle-shaped cells aggregated around the (A) BCP and (B) SFP scaffolds were observed. Original magnification, ×100. H&E, hematoxylin and eosin; BCP, biphasic calcium phosphate ceramic; SFP, silk fibroin protein matrix.

Images representative of the H&E staining in the BCP and SFP groups are shown in Fig. 3. The BMSCs encapsulated in the BCP and SFP were sparse and spindle-shaped, with almost no characteristics of chondrocytes. As for the degradation of the scaffold, it was more marked in the SFP group than in the BCP group. As no tissue was identified in the CS group, no H&E staining results were obtained.

**Figure 4:**
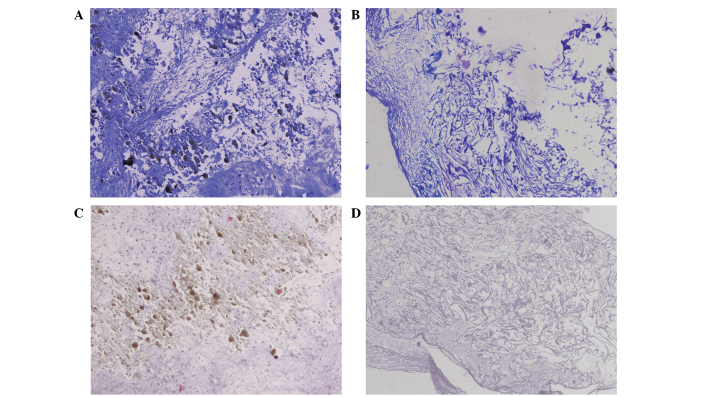
(A and B) Toluidine blue staining and (C and D) immunohistochemical staining of the sectioned tissue. The tissue from the (A) BCP and (B) SFP scaffolds did not stain metachromatically, indicating no glycosaminoglycan deposition. No staining for type II collagen was observed for the tissue from the (C) BCP and (D) SFP scaffolds. Original magnification, ×100. BCP, biphasic calcium phosphate ceramic; SFP, silk fibroin protein matrix.

As reflected in the toluidine blue staining assay, no metachromatic staining of the cell matrix was observed, indicating that that minimal proteoglycan and glycosaminoglycan deposition was present in the BCP and SFP groups (Fig. 4A and B). In agreement with the toluidine blue staining, the immunohistochemical staining of type II collagen (Fig. 4C and D) indicated the absence of stained type II collagen for all the constructs in the BCP and SFP groups. These results suggest that the BMSCs did not undergo chondrogenic differentiation when loaded in the BCP or SFP.

## Discussion

The present study showed that the BCP, SFP and CS did not initiate chondrogenic differentiation of the BMSCs *in vivo*. The histological and immunohistochemical examinations revealed cells with no characteristics of the chondrocyte phenotype and there was little expression of aggrecan type II presented in the BMSC-BCP and -SFP constructs. No tissue was present in the constructs with CS as the scaffold. The results indicate that no chondrogenic differentiation of BMSCs occurred when BCP, SFP and CS were used as scaffolds.

Regarding a previous study in which it was shown that chondrogenesis is induced by collagen and collagen-alginate hydrogels, the present study further demonstrated that chondrogenesis by materials is material-dependent. In the previous study, collagen-based hydrogels were demonstrated to have the ability to induce chondrogenesis *in vivo* without the addition of any growth factors. This indicated that hydrogels, instead of porous materials such as BCP, SCF and CS, may provide a more favorable environment for chondrogenesis, mimicking that of natural cartilage (8,9). The result was in accordance with the study by Fujisato *et al* which demonstrated that a surface layer of cells seeded in porous materials easily leads to a change in the phenotype of the cells to flat and amebocyte- or fibroblast-like (10).

However, the results revealed that the diffusion chamber system was useful in allowing nutrition and body fluids to pass through while preventing invasion by host cells, as previously observed (11). It is of significance for chondrogenesis that vascular invasion, which may lead to calcification and bone formation through the process of endothelial ossification, was effectively avoided by the filter membrane.

Based on previous findings of chondrogenic induction by collagen-based hydrogels, the present study provides further evidence to indicate that chondrogenic induction by materials is material-dependent. Hydrogels are superior to porous materials in the induction of chondrogenic differentiation.
